# Plasma Proteomic Signatures for Alzheimer's Disease: Comparable Accuracy to ATN Biomarkers and Cross‐Platform Validation

**DOI:** 10.1002/acn3.70227

**Published:** 2025-10-13

**Authors:** Manyue Hu, Oliver Robinson, Christina M. Lill, Anna Matton, Raquel Puerta, Pilar Sanz, Merce Boada, Agustín Ruiz, Lefkos Middleton, the Alzheimer's Disease Neuroimaging Initiative

**Affiliations:** ^1^ Ageing Epidemiology (AGE) Research Unit, School of Public Health Imperial College London London UK; ^2^ MRC Centre for Environment and Health, Department of Epidemiology and Biostatistics, School of Public Health Imperial College London London UK; ^3^ Institute of Epidemiology and Social Medicine University of Münster Münster Germany; ^4^ Department of Neurobiology, Care Sciences and Society Karolinska Institutet Stockholm Sweden; ^5^ ACE Alzheimer Center Barcelona Universitat Internacional de Catalunya Barcelona Spain; ^6^ CIBERNED Network Center for Biomedical Research in Neurodegenerative Diseases, National Institute of Health Carlos III Madrid Spain; ^7^ Glenn Biggs Institute for Alzheimer's & Neurodegenerative Diseases University of Texas Health Science Center San Antonio Texas USA; ^8^ Department of Microbiology, Immunology and Molecular Genetics, Long School of Medicine University of Texas Health Science Center San Antonio Texas USA

**Keywords:** Alzheimer's disease, cognitive dysfunction, cohort study, machine learning, proteomics

## Abstract

**Background:**

There is growing recognition of the potential of plasma proteomics for Alzheimer's Disease (AD) risk assessment and disease characterization. However, differences between proteomics platforms introduce uncertainties regarding cross‐platform applicability.

**Objective:**

We aimed to identify a detailed plasma biosignature for distinguishing AD from cognitively normal (CN) and another signature for classifying mild cognitive impairment (MCI) decliners and non‐decliners. We also explored the cross‐platform applicability of these models between two proteomic platforms.

**Methods:**

Elastic net was performed on 190 plasma analytes measured using the Luminex xMAP platform in 566 participants from the Alzheimer's Disease Neuroimaging Initiative (ADNI) to model MCI stable/decliner and AD/CN classification. MCI decliner was defined as progression to AD during follow‐up (mean 4.2 ± 3.2 years). External cross‐platform validation was conducted with 1303 participants from the Spanish Ace study, using the SOMAscan 7k platform.

**Results:**

An 11‐analyte signature for distinguishing AD from CN achieved a 93.5% accuracy on ADNI and 95.2% on Ace. The ApoE and BNP proteins were the two most important contributors to the classifier. The MCI classification signature performed less well, with 65.9% accuracy on ADNI and 51.0% accuracy upon validation testing in Ace.

**Discussion:**

Compared with prior proteomic‐based studies on the same dataset, our findings attained higher specificity and sensitivity for AD classification while utilizing a smaller panel of analytes. We also confirmed the reliability and consistency of this signature within a different population from a different platform. The plasma proteomic platforms explored were, however, not sufficient to determine MCI decliners versus non‐decliners.

## 1 Introduction

Alzheimer's disease (AD) is a relentlessly debilitating neurodegenerative disease, characterized by slowly progressive cognitive and functional decline [1] that gradually leads to a lack of ability to perform the simplest tasks in daily life. Mild Cognitive Impairment (MCI) due to AD is the first detectable clinical disease stage of AD [2]. MCI is typically preceded by a long pre‐clinical period of progressive accumulation of the AD pathological AT[N] (ATN) triad signature [3] of extracellular amyloid‐β (Aβ) plaques, intracellular tau‐enriched neurofilament tangles (NFT), and neurodegeneration features. Clinical MCI symptoms become noticeable once these pathologies reach abnormally high levels [4].

The MCI stage can potentially endure for several years, and some patients may oscillate, revert, or remain stable for a variable period of time [5], although they remain at an elevated risk of progressing to dementia [6]. However, despite the elevated risk of AD among patients with MCI compared to cognitively normal elderly individuals [7, 8], numerous studies indicate that some individuals diagnosed with MCI may revert to normal cognition, sometimes following changes in lifestyle, better management of co‐morbidities, or even spontaneously [8, 9, 10]. Thus, the biomarker‐based early and accurate diagnosis of MCI, along with the ability to predict progression patterns towards further cognitive decline vs. relative stability or reversal of MCI decliners, may help in enhancing effective personalized management of patients and their treatment outcomes and contribute towards alleviating pressures on healthcare systems.

The emphasis on early or pre‐symptomatic diagnosis is further supported by results from recent randomized controlled trials (RCTs) of two anti‐amyloid immunotherapies [11, 12], which demonstrated maximal benefits in early MCI patients, while trials are also underway to assess these therapies in pre‐symptomatic individuals at high risk for AD. It is widely acknowledged that an ideal biomarker should not only exhibit stability in terms of functionality, specificity, sensitivity, and accuracy but also possess simplicity, preferably allowing direct measurements in easily accessible standard biological sources such as plasma, serum, saliva, or urine [13, 14].

The Alzheimer's Disease Neuroimaging Initiative (ADNI) [15] has been running since 2004, with the overarching goal of gaining a deeper understanding of the biological mechanisms underlying MCI and AD. Over nearly two decades, ADNI has made a wide range of clinical, cognitive, and genetic data collected across five different phases (ADNI1, ADNI2, ADNI3, ADNI4, and ADNI‐GO) publicly available. Prior studies, based on ADNI and other cohort studies, have identified plasma protein‐based signatures, using the Luminex xMAP assay, capable of differentiating between AD, cognitively unimpaired or normal individuals (CN), and MCI [16, 17, 18]. Recent advancements in blood‐based biomarkers (BBMs) for AD, such as the use of ptau217 and Aβ_42_/Aβ_40_ in clinical trials for screening, indicate the promise of BBMs in clinical settings [19]. The integration of proteomic signatures, either alone or in combination with these BBMs, holds promise for enhancing the diagnostic yield of both clinical and pathological diagnoses and may also help distinguish potential AD biotypes [20].

Due to rapid advancements in biotechnology, new methods for biomarker assays continue to expand at an accelerated pace. As an emerging high‐throughput and large‐scale protein quantification method, SOMAscan is gaining popularity, but it has primarily been evaluated in comparison with platforms like Olink [21], with limited attention to its transferability in relation to some broadly used platforms, such as Luminex xMAP technology. Given that multiplex immunoassays have long been used in proteomics studies to measure biomolecular interactions on a high‐throughput, high‐content platform [22], further investigation into the transferability and limitations of the SOMAscan and other emerging assays, compared with traditional multiplex immunoassays, would aid in coherently understanding proteomic patterns in dementia studies. Cross‐platform correlation of protein measurements remains unclear for some technologies [23, 24], and for some technologies it remains a challenge. While most studies assessing cross‐platform transferability focus on evaluating protein measurement correlations [25, 26], few have directly validated transferability by building predictive models based on data from one platform and testing them on another.

In this study, we aimed to develop a method of selecting and utilizing a small protein panel beyond the core AD pathology for increased diagnostic specificity and sensitivity of AD and another panel that allows differentiation of MCI patients who progress to dementia stages from those who remain stable or revert, using targeted panels of multiplex assays conducted in ADNI. Furthermore, we aimed to validate the robustness of our models and signatures across a different platform and population by testing the two models on an independent sample, the Ace CSF cohort, using SOMAscan data.

## 2 Methods

### 2.1 Study Population: ADNI Dataset

The proteomic data used in this study were the dataset “Biomarkers Consortium Plasma Proteomics Project RBM multiplex data” derived from the Biomarkers Consortium “Use of Targeted Multiplex Proteomic Strategies to Identify Plasma‐Based Biomarkers in Alzheimer's Disease” project, a subset from ADNI. This dataset encompasses 190 plasma analytes, collected from 566 participants at baseline and 12 months after, chosen based on their relevance for deciphering diseases such as cancer, cardiovascular diseases, metabolic disorders, and inflammation. Details are available in the ‘Biomarkers Consortium ADNI Plasma Targeted Proteomics Project‐Data Primer’ file [27]. The demographic data and baseline clinical assessment were obtained accordingly from the ADNI database (http://adni.loni.usc.edu/).

All the characteristics of the study population were compared using one‐way analysis of variance (ANOVA).

### 2.2 Diagnostic and Classification Criteria

The 566 subjects for this study were classified at baseline as CN (*n* = 58), prevalent MCI (*n* = 396), or prevalent AD (*n* = 112) by ADNI based on their diagnosis protocol [28]. According to the ADNI diagnosis summary, in terms of memory complaints, CN subjects exhibited none, whereas both MCI and AD participants were required to have such complaints. The MMSE scores ranged from 24 to 30 for both CN and MCI subjects, and from 20 to 26 for AD patients. CDR scores were set at 0 for CN individuals and 0.5 for MCI participants, with a requirement that their memory box score be at least 0.5. AD subjects scored between 0.5 and 1. For memory assessment, delayed recall from one paragraph of the Logical Memory II subscale of the Wechsler Memory Scale–Revised (maximum score: 25) was used. The cutoff for normal subjects varied by education level: scores of 9+ for 16 years of education, 5+ for 8–15 years, and 3+ for 0–7 years. For MCI and AD participants, cutoff scores were 8 or below for 16 years of education, 4 or below for 8–15 years, and 2 or below for 0–7 years [28].

Based on a follow‐up of up to 198 months (average 4.19 ± 3.21 years), we categorized the MCI participants into MCI reverters (reverted to CN and maintained until follow‐up ended, *N* = 21), MCI stable (consistently diagnosed as MCI or occasionally as CN or AD but back to MCI before follow‐up ended, *N* = 158), and MCI decliners (declined and remained as AD dementia until follow‐up ended, *N* = 217).

### 2.3 Data Pre‐Processing

The plasma analyte levels of all participants were quantified using the Human Discovery Map panel developed by Rules‐Based Medicine (RBM) on the Luminex xMAP platform [29]. Seventy participants' values were missing at month 12. Outliers (> 5 SD) were replaced with the nearest non‐outlier values. As part of the Biomarkers Consortium project [29], analyte distributions within each diagnosis group were transformed to approximate normality (using Box‐Cox transformations [30]) and then z‐scored. Ultimately, 146 of the 190 analytes passed quality control and were included in our analysis (Table S1).

### 2.4 Data Modeling

Several models were considered for our analysis, based on previous relevant publications [16, 18, 31, 32, 33]. We employed a two‐step process to generate our final models: first, features were selected as detailed below, and then classifiers were applied to the selected feature sets.

### 2.5 Feature Selection and Dimension Reduction

In our initial feature selection step, and to provide an overview of single analyte associations with AD status, logistic regression was conducted for each analyte, with covariate adjustments (age, sex, *APOE* ε4 genotype [rs429358]), to assess their associations with AD patients and CN participants. Multiple testing was corrected for using a Bonferroni correction for the effective number of tests, according to the correlation matrix of all analytes from the PoolR package [34].

We applied the elastic net approach together for its proven efficacy in handling multicollinearity, as a compromise [35] between the two regression methods, LASSO and Ridge. Of note, elastic net has been gaining increasing popularity by researchers in AD proteomics for feature selection and classifier development [36, 37]. We also employed random forest [38] and naïve bayes [39], as these machine learning algorithms have consistently demonstrated effectiveness in distinguishing between AD patients and CN individuals based on proteomic data [17, 40, 41, 42]. All analytes and covariates (age, sex, *APOE* ε4 genotype) were used in the model training.

Aimed at optimizing feature selection and model stability, random forest and elastic net were each run 100 times on the AD/CN subset. Analytes appearing in over 50% of random forest models and in all elastic net models were selected for classification (Figure S1).

### 2.6 Classification

For AD and CN participants classification, we applied random forest, naïve bayes, and elastic net from the caret package in R [43], using the features selected, to classify AD and CN participants. These three methods were applied to signature LR, EN, RF, and CM, to build 12 models.

For MCI subgroup classification, only elastic net was used for feature selection, dimension reduction, and classification, as it is the model with the best performance in the above‐mentioned AD versus CN classification.

### 2.7 Model Testing

Considering the relatively small sample size, we validated models using a cross‐validation approach: By randomly splitting the 170 participants (112 AD and 58 CN) into training (*N* = 136) and testing (*N* = 34) sets for 10 times, we trained and tested the classifiers each time and calculated their mean accuracy, specificity, and sensitivity across the 10 datasets. The selected model was also applied to the follow‐up data at month 12 for temporal validation and to the MCI dataset for exploring the signatures' ability to distinguish MCI stable and decliners.

Analyses were performed in R (version 4.3.2) with the key packages glmnet (v4.1.8) and caret (v7.0.1).

### 2.8 Model Validation: The Ace Cohort

The Ace CSF cohort dataset was used as an independent validation cohort in this study. Ace Alzheimer Centre Barcelona (www.fundacioace.com/en) was founded in 1995 as a centre of research and clinical excellence in Alzheimer's disease and related dementias [44, 45]. The Ace Alzheimer Center Barcelona has established a comprehensive cohort aimed at advancing the understanding, diagnosis, and treatment of Alzheimer's disease (AD) and other dementias. This cohort serves as a resource for research initiatives, particularly in biomarker discovery and validation [46, 47, 48], for cerebrospinal fluid (CSF) AD biomarkers [49], plasma‐based biomarkers [50, 51] and also in exploring genetic aspects in late onset dementia [52].

The Ace subset used in this study contains 1303 participants (113 CN/795 MCI/395 prevalent AD), was derived based on the selection of protein data included in the final model from ADNI. The syndromic diagnosis of all subjects was established by a multidisciplinary group of neurologists, neuropsychologists, and social workers, based on their clinical protocols [46, 49, 53]. Plasma samples [54] was obtained following the consensus recommendations [51, 54], and a subset of these were analyzed using the SOMAscan 7k proteomic platform (SomaLogic, Boulder, CO, US) and processed according to standard Somalogic procedures. 7307 plasma analytes were available, including all the classification analytes that were selected from the ADNI cohort.

Pre‐processing (outlier treatment, transformation, and scaling) was conducted using the same steps used in ADNI (described above).

The analyte plasma levels identified from the ADNI dataset, along with covariates (age, sex, and *APOE* ε4 status), were applied to the Ace cohort. The classifiers' accuracy was evaluated by comparing their predictions to actual diagnostic categories or MCI subgroups, assessing their precision in distinguishing among these groups.

## 3 Results

### 3.1 Baseline Demographic Characteristics

In ADNI dataset, there were no significant differences of age, sex, and education between diagnosis groups. Significant differences were shown for *APOE* ε4 genotype (*p* < 0.001) and MMSE (*p* < 0.001) between diagnosis groups. For MCI subgroups, no significant difference was observed (*p* > 0.1 for all features) (Table 1).

**TABLE 1:** Characteristics of participants.

(a)				
Diagnosis	CN	MCI	AD	*p*
Number	58	396	112	
Age (range)	75.3 (62–90)	74.9 (54–90)	75.4 (54–89)	0.728
M/F (baseline)	30/28	256/140	65/47	0.838
AopEε4	8.60%	53.30%	67.90%	< 2e^−16^
Education (range)	15.67 (8–20)	15.64 (4–20)	15.12 (4–20)	0.06
MMSE ± STD	28.93 ± 1.15	27.03 ± 1.78	23.59 ± 1.92	< 2e^−16^
ADAS ± STD	9.61 ± 4.15	18.64 ± 6.27	28.53 ± 7.77	< 2e^−16^

(b)				
MCI	MCI stable	MCI decliner	MCI reverter	*p*
Number	158	217	21	
Age (range)	75.3 (56–89)	74.8 (55–89)	72.0 (54–90)	0.12
M/F (baseline)	101/57	138/79	17‐Apr	0.28
AopEε4	44.30%	63.10%	19.00%	0.33
Education (range)	15.36 (4–20)	15.82 (6–20)	15.90 (12–20)	0.22
MMSE ± STD	27.20 ± 1.82	26.85 ± 1.74	27.48 ± 1.75	0.1
ADAS ± STD	17.09 ± 6.04	20.46 ± 5.80	11.89 ± 5.04	0.39

*Note:* *p* Value was calculated with analysis of variance (ANOVA).

Abbreviations: AD, Alzheimer's disease; CN, cognitive normal; MCI, mild cognitive impairment; STD, standard deviation.

### 3.2 Feature Selection for AD/CN Classification

**FIGURE 1:**
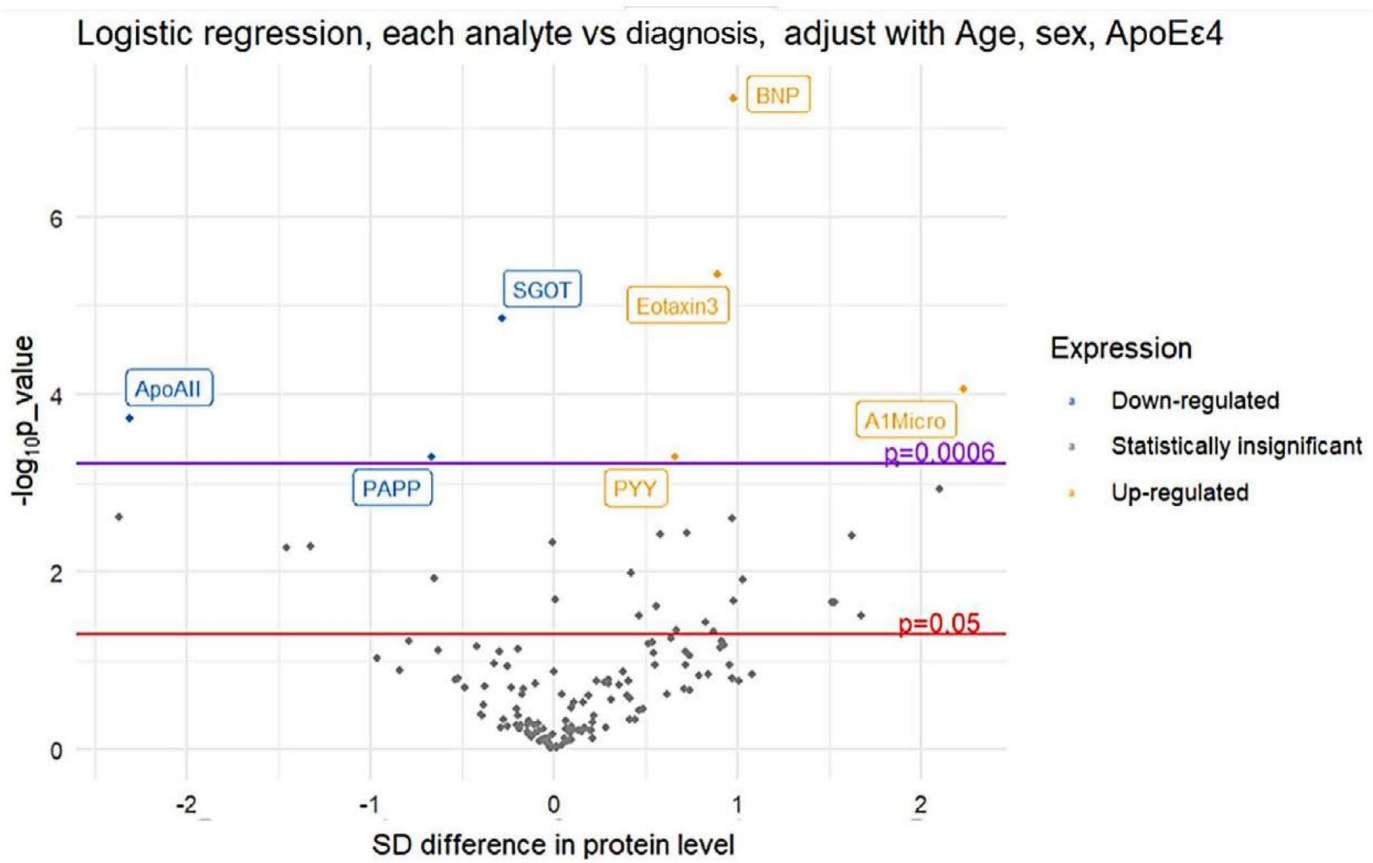
Volcano plot of analyte associations with diagnosis (AD/CN). *p* Value threshold was corrected using a Bonferroni correction for the effective number of tests.

Covariate‐adjusted (age, sex, *APOE ε4* genotype) logistic regression was used to assess single‐analyte associations with AD diagnosis. The 7 analytes whose *p*‐value passed Bonferroni correction (alpha = 0.0006) were selected for signature LR (Figure 1).

Fifteen and eleven analytes were selected by elastic net and random forest for signature EN and signature RF respectively. Signature CM contained four common analytes of signature LR, EN, and RF. Signature CV only included covariates to assess whether the inclusion of additional analytes enhances classifier performance (Table 2). Different classifiers based on these features were then applied, and the mean accuracy across 10 randomly generated test sets of different models was compared. The signature EN and elastic net classifier gained the highest accuracy and the strongest performance (Table S2).

**TABLE 2:** Signatures selected by different methods.

Signature 1 LR	Signature 2 elastic net	Signature 3 random forest	Signature 4 common	Signature 5 covariates
(*N* = 7)	(*N* = 15)	(*N* = 11)	(*N* = 4)	(*N* = 3)
A1Micro	IL16	Eotaxin3	ApoAII	Age
PYY	ApoAII	BNP	BNP	Sex
ApoAII	ApoB	SGOT	SGOT	APOEε4 status
PAPP	ApoE	ApoAII	PYY	
BNP	Vitronectin	ApoE		
Eotaxin3	BNP	A1Micro		
SGOT	IL6r	BTC		
	TTR	PLGF		
	PYY	PYY		
	SGOT	PAPP		
	CEA	IGM		
	MIGI			
	MIP1a			
	ICAM1			
	IP10			

*Note:* LR refers to LR. ApoE refers to the plasma level of the ApoE protein, while APOEε4 status refers to the APOE ε4 genotype.

### 3.3 Dimension Reduction of Elastic Net‐Selected Signature

While elastic net selection varies across runs, consistent analytes emerged when panel size was fixed (Figure S1). Based on their occurrence frequency and performance of panels across test sets, an 11‐analyte panel (ApoAII, ApoB, IL16, ApoE, Vitronectin, BNP, TTR, IL6r, PYY, SGOT, and MIP1a) was selected as the final signature due to its highest occurrence frequency and high accuracy on AD vs. CN classification (Table S3). Across the same 10 test sets, the 11‐analyte signature had a higher average accuracy (86.5% vs. 86.3%) and specificity (82.9% vs. 81.1%) on AD vs. CN classification than the above‐mentioned 15‐analyte signature, but a slightly lower sensitivity (90.1% vs. 91.5%). Compared to the covariate‐only signature (accuracy 74.4%), the 11‐analyte signature (86.5%) also substantially improved disease classification.

### 3.4 Validation of 11‐Analyte Signature on 12‐Month Follow‐Up

To assess how accurate our 11‐analyte signature was when applied to repeat biomarker measurements (which may vary over time), we applied the model to 247 ADNI participants who were diagnosed as AD/CN and had plasma analyte levels measured 12 months later. The accuracy was 72.60% (sensitivity 81.65%, specificity 49.18%), indicating the influence of temporal variation on the accuracy of the classifier.

### 3.5 MCI Decliners Versus MCI Stables

The 11‐analyte model classified MCI decliners versus stables with 59.2% accuracy, outperforming the covariate‐only model (53.9%). Then we explored whether developing a biosignature specific for MCI conversion could improve accuracy. Elastic net feature selection and classification (as described above) trained on MCI participants resulted in a 17‐analyte signature (Table S4). The accuracy of the 17 analyte was 58.9%, thus did not show an improvement compared to the 11‐analyte model.

### 3.6 Validation of Signatures on the Ace Cohort

In the Ace study, there are significant differences in terms of age (*p* < 0.001), sex (*p* = 0.004), and *APOE ε4* gene carrier status (*p* = 0.016) between diagnostic groups. For MCI decliners vs. stable, significant differences can still be observed in age (*p* < 0.001) and *APOE ε4* status (*p* < 0.001), but not sex (*p* = 0.15). The mean follow‐up time for the 795 MCI participants was 2.6 years, with a standard deviation of 1.6 years. None of the MCI patients reverted to CN status during follow‐up (Table 3), which may be attributed to differences in ascertainment methods. Unlike ADNI, where participants were recruited through broader community‐based strategies, the Ace CSF cohort was more selectively composed of individuals seeking medical attention, potentially leading to a lower likelihood of identifying MCI reverters.

**TABLE 3:** Characteristics of participants from Ace cohort.

(a)				
Ace diagnosis	CN	MCI	AD	*p*
Number	113	795	395	
Age (range)	66.6 (51–82)	72.9 (47–93)	74.8 (38–92)	< 2e^−16^
M/F (baseline)	46/67	364/431	139/256	0.004
AopEε4	23.10%	34.40%	37.50%	0.016

(b)			
Ace MCI	MCI stable	MCI decliner	*p*
Number	449	346	
Age (range)	71.0 (47–90)	75.4 (52–93)	< 2e^−16^
M/F (baseline)	211/238	153/193	0.15
AopEε4	37.40%	43.40%	6.00E−08

*Note:* *p* Value was calculated with analysis of variance (ANOVA). (a) Characteristics of all participants. (b) Characteristics of MCI participants.

When comparing with ADNI, significant characteristic differences exist between the two cohorts (age, sex, *APOE ε4* status, all *p*‐values < 0.001), as well as varying proportions of AD and CN participants (66% AD in ADNI and 78% in Ace). The mean follow‐up time is also different (4.2 vs. 2.6 years). When applying the covariate‐only signature model generated from ADNI to the Ace cohort for predicting MCI classification, a low accuracy (46.7%) was observed.

We first employed the same data pre‐processing steps as those in ADNI for Ace cohort data pre‐processing. Subsequently, the 11‐analyte signature model was applied to distinguish between the AD and CN groups and achieved an accuracy of 95.2% (specificity 96.5%, sensitivity 92.7%), which is much higher than the covariates‐only signature (accuracy 46.7%) (Table 4). The low accuracy of the covariates‐only signature may be attributed to differences in covariate distributions between the two datasets. When comparing the AD and CN cohorts from Ace with ADNI, Ace has significantly younger CN participants, a lower male/female sex ratio, and fewer *APOE ε4* allele carriers (all *p*‐values < 0.0001).

**TABLE 4:** Model performance on ADNI and Ace cohort.

(a)				
AD/CN classification	Covariates‐only accuracy	11‐analyte accuracy	11‐analyte specificity	11‐analyte sensitivity
ADNI	74.38%	93.53%	87.93%	93.75%
Ace	46.65%	95.19%	96.46%	92.66%

(b)				
MCI classification	Covariates‐only accuracy	17‐analyte accuracy	17‐analyte specificity	17‐analyte sensitivity
ADNI	53.88%	65.87%	39.87%	84.79%
Ace	43.52%	51.03%	35.63%	68.21%

*Note:* Covariates only accuracy: Classification accuracy on ADNI or Ace cohort. 11‐analyte: The 11 analyte and covariates model for AD/CN classification, 17‐analyte: The 17 analyte and covariates model for MCI classification.

We also utilized the 17‐analyte signature generated from ADNI MCI classification for the identification of MCI stable and MCI decliners. The accuracy was only 51.0%, but it was still higher than the covariates‐only signature (43.5%). Despite the significant difference in cohort characteristics, this indicates that analytes may provide additional information beyond covariates, although the performance remains limited.

### 3.7 Comparison of Classifier‐Selected Analyte in ADNI and Ace

**FIGURE 2:**
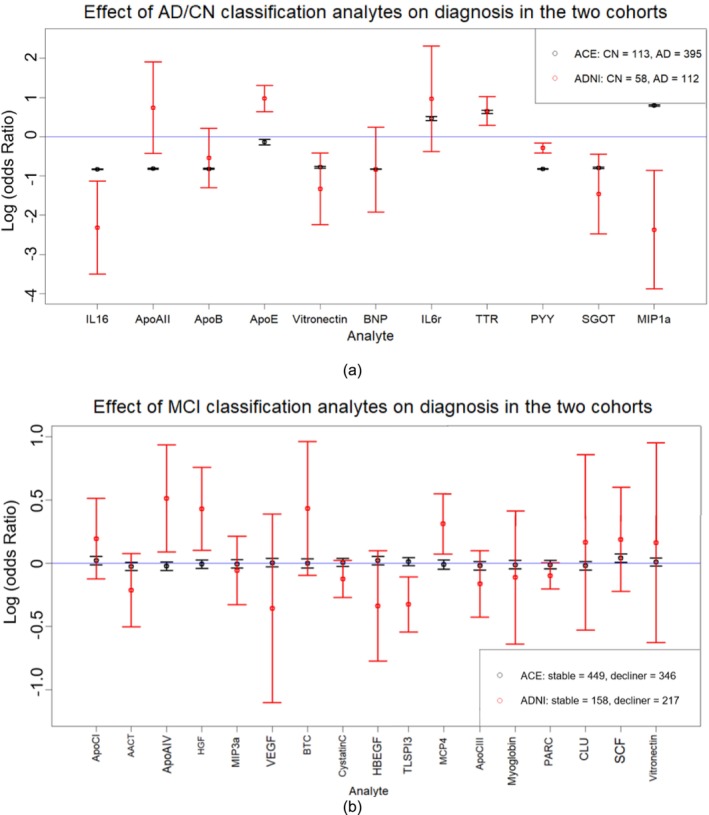
Effect of analytes on classification in different cohorts. Figure a displays the 11 analytes selected for AD/CN classification, while figure b refers to the 17 analytes used for MCI classification. The units of the log(OR) is per SD of analyte. Effect on diagnosis and classification was calculated using covariate‐adjusted logistic regression models for each analyte individually. Black refers to the results from Ace cohort, while red refers to those from ADNI study. The length of the error bars reflects the 95% confident interval.

To account for cohort and platform differences between ADNI and Ace, we assessed their impact on analyte‐diagnosis associations (Figure 2). Thus, we employed covariate‐adjusted logistic regression to analyze the association of each analyte separately with diagnosis, in both ADNI and Ace cohorts. Eight of the 11 analytes showed the same directions of effect across cohorts (Figure 2a). BNP, PYY, and ApoB confer comparable effect sizes, and their contributions to the classification model are relatively high. While MIP1a, ApoAII, and ApoE showed opposite directions of effect, their contribution to the classification model was smaller in comparison to that of the others (Table S3). These discrepancies may stem from differences in measurement methodologies between the SOMAscan and RBM platforms, as previous studies have reported lower correlation for some proteins across these technologies [25]. Overall, 7 out of 11 analytes in ADNI and all analytes in Ace exhibited *p*‐values less than 0.05 in these univariate analyses, indicating their significance in distinguishing between AD and CN.

The log odds ratios of 17 analytes used for classifying MCI stable and MCI decliners display substantial differences across the two cohorts (Figure 2b). Eight of the 17 analytes show opposite directions of effect, and their contributions to the model are relatively higher than other analytes (Table S4). Additionally, the relatively large standard errors—which consistently include zero—and the limited number of analytes with *p*‐values below 0.05 (6 in ADNI and 1 in Ace) could further contribute to this reduced accuracy.

## 4 Discussion

In this study, we have developed a classification model containing 11 plasma biomarkers and covariates (sex, age, and *APOE ε4* genotype), and hereby demonstrate a cross‐validated prediction accuracy of AD and CN classification around 94% with the sensitivity and specificity of 94% and 88%, within the ADNI cohort. Recent studies show plasma p‐tau217 and ATN‐based markers (CSF Aβ42/Aβ40, tau‐PET) achieve AUCs > 0.9 for AD diagnosis [55]. Similarly, ATN‐based classification in the ADNI cohort, using CSF Aβ42/Aβ40 (AUC = 0.84) and tau‐PET (AUC = 0.92), also demonstrated strong performance [56]. Our model—despite not incorporating ATN plasma biomarkers—achieved comparable accuracy, suggesting that this set of plasma analytes and covariates may offer an additional exploratory perspective for a better understanding of biological mechanisms underlying the development of AD and, thus, help identify novel biological pathways or systemic processes relevant to disease development and progression. Furthermore, when applied to the independent validation Ace cohort, the performance of the 11‐analyte model had an accuracy of 95%, despite the analytes being measured through a different analytical proteomic platform. However, the MCI classification model, containing covariates and 17 analytes, performed less well, achieving a 10‐fold cross‐validated accuracy around 66% (specificity 40%, sensitivity 85%) in the ADNI cohort and did not show any predictive accuracy in the Ace cohort (51%).

Our AD/CN classification model, developed on analytes measured at baseline, exhibited diminished performance when applied to the month 12 measurements in ADNI, dropping to 73.6%. This limited temporal stability of the signature might be attributed to the fact that the concentration of substances in plasma is influenced by a variety of modifiable factors such as lifestyle, nutrition, and infections [57, 58]; and should, therefore, not be solely attributed to alterations in brain pathophysiology. The model may also be overfitted to baseline patterns, limiting its generalizability over time. However, some individuals classified as CN clinically may harbor brain pathology consistent with more advanced AD clinical stages; thus, the model may indeed detect the true “biological signals” [59, 60].

The MCI classification model achieved, in the ADNI cohort, a cross‐validated accuracy of 58% in distinguishing MCI decliners from MCI stable. When applying the 17‐analyte model to the Ace cohort, the accuracy was only 51%. This poorer performance may be related to the nosological heterogeneity of MCI, the influence of unaccounted confounding factors, and/or the presence of overlapping biological processes between MCI stable and decliners. Recent studies also suggest that plasma biomarkers alone may offer limited utility in distinguishing progressive from stable MCI. Reported AUCs for predicting MCI‐to‐AD conversion using plasma‐based models typically range from 0.64 to 0.70 [61, 62], indicating suboptimal performance for clinical application. In contrast, transcriptomic signatures from peripheral blood have achieved higher accuracy (AUC > 0.90) in differentiating progressive from stable MCI [63], and multimodal approaches incorporating neuroimaging or genomics may offer more robust alternatives [64]. Meanwhile, even identifying MCI from AD or CN remains challenging across different biomarker‐based models. While plasma‐based ATN biomarkers significantly improved AD conversion prediction over a basic model (age, sex, education, AUC = 0.64), even the best‐performing model incorporating multiple plasma biomarkers achieved an AUC of only 0.82 [56], suggesting that plasma biomarkers alone may not fully capture the heterogeneity of MCI both for identifying MCI from AD or CN and subtyping MCI. Additionally, the lack of discernible differences in covariates between the two groups within the ADNI dataset also resulted in diminished accuracy of classification models. Consequently, when this model is applied to categorize a new cohort with analogous analyte distributions but disparate covariate distributions, the accuracy is likely to be further compromised. Additionally, when comparing MCI subgroups based on a follow‐up period of up to 48 months (mean 2.8 ± 1.5 years) in ADNI, comparable to the Ace cohort, to those based on up to 198 months in ADNI participants, we observed that 10.9% of participants shifted between MCI subgroups, notably among MCI stable and reverters. This suggests that participant classifications in the Ace cohort may not fully capture their cognitive trajectories, potentially affecting the accuracy of MCI decliner and stable classifications in our study.

### 4.1 Common Analytes in Different Signatures

In this study, vitronectin was the only analyte found to be a potential predictor both of AD and CN status and progression of MCI to dementia. Despite its lower coefficient, vitronectin, a regulator of the extracellular environment [65], has been implicated in inflammation and amyloid‐related deposition [66, 67], though its role in AD remains unclear.

The limited overlap between the AD‐CN signature and MCI signatures may stem from multiple factors. Crane et al. [68] noted statistically significant differences in measurement precision across cognitive domains, and others suggested that 34.2% of MCI were “false positive” (CN clinically misclassified as MCI) and 7.1% were false negatives (MCI misclassified as CN) within the ADNI dataset [69, 70], In spite of these observations, we adopted the ADNI “formal” diagnosis for our analyses. Furthermore, the analytes selected for the AD‐CN signature reflect markers of advanced disease stages that may not be detectable in MCI patients, especially those in the early stages or with minimal cognitive decline.

### 4.2 Comparison With Previous Work on This ADNI Subset

Several researchers have previously attempted to classify AD and CN participants in ADNI, using plasma proteomic signatures [16, 17, 18]. Compared with prior studies, our signature attained higher specificity and sensitivity using the same dataset, albeit using a smaller panel. Additionally, our model was validated on an independent cohort utilizing a different proteomics platform, further corroborating the robustness and generalizability of our findings.

Among the components of our and prior proteomic signatures [16, 17, 18] for AD‐CN classification, the common proteins are ApoE (Apolipoprotein E) and BNP (B‐type natriuretic peptide). Previous studies have suggested that the ability of plasma ApoE to distinguish between AD and CN individuals is primarily driven by *APOE ε4* genotype [71]. However, in our analysis, ApoE was still selected even after adjusting for APOE genotype, although its coefficient in the model was lower than the average. This could be due to the residual predictive value of ApoE beyond genotype or the population heterogeneity. ApoE is a lipid transporter which is involved in various cellular functions, such as neural signal transmission and neuroinflammation [72]. BNP primarily regulates circulation and vascular function. The exact mechanism relationship between BNP and AD, as well as its potential as an AD biomarker, warrants further future research [73, 74].

### 4.3 High Classification Effect Analytes

In the 11‐analyte classification model, distinguishing between AD and CN, BNP was found to exhibit the greatest contribution and exhibited similar and significant association with AD diagnosis in both ADNI and ACE cohorts. Although the mechanism still remains unclear, there have been several reports of an association between elevated BNP levels and increased AD risk, both in in vivo cohort studies [74] and post mortem neuro‐pathological studies [73]. For MCI classification, myoglobin was the analyte with the strongest effect in our study. Myoglobin was one of the few analytes to replicate the association with MCI classification in Ace. It is a cytoplasmic hemoprotein capable of reversibly binding oxygen (O_2_) and releasing it during periods of hypoxia or anoxia [75]. It may serve as a scavenger of reactive oxygen species (ROS) within cells [76], mitigating oxidative stress. Numerous clinical trials [77, 78, 79] have shown that altering brain oxygenation intermittently can effectively enhance short‐term memory and attention in elderly individuals with amnestic MCI (aMCI) [80] and improve cognitive function [81, 82].

### 4.4 Strengths and Limitations of Our Work

Our signature is based on elastic net, a statistical modeling and machine learning method widely used for high‐dimensional data analysis. Its feature selection process is entirely data‐driven to comprehensively capture all relevant analytes associated with AD [83, 84]. Given the ubiquitous correlations among human genetic products [85], elastic net can effectively mitigate the issues arising from collinearity among analytes by employing L1‐norm penalty and has the additional advantage of generating sparse models with the most influential features. Additionally, dimensionality reduction reduced panel size with minimal impact on accuracy. External validation in the Ace cohort further supported the model's generalizability across platforms. However, our model exhibited lower performance in classifying MCI converters using a similar approach. Limitations include outdated analyte selection, diagnostic variability, and limited platform validation. Future studies should expand analyte coverage, harmonize diagnostic criteria, and explore cross‐platform reproducibility.

In summary, our results confirm the reliability and consistency of a group of plasma analytes across different platforms and their potential utility in contributing to a more precise and personalized characterization of AD individuals. This may prove of value in future pharmaceutical research and development efforts, towards more effective and personalized new therapies for cognitive decline and dementia due to AD. In this study, using elastic net, we have developed and externally validated a precise and sensitive biomarker signature for AD identification, which may potentially have value as an additional plasma biomarker‐based and data‐driven tool in classifying disease status. We further highlight challenges in transferring these AD protein‐based signatures to MCI classification. Our findings underscore the importance of refining diagnostic criteria, considering heterogeneous disease characteristics, and assessing cross‐platform consistency when developing plasma‐based classification models for AD and MCI. Future studies should explore more standardized diagnostic frameworks and validate models across multiple assay platforms to enhance the sensitivity and robustness of biomarker‐based classification.

## Author Contributions

**Manyue Hu:** conceptualization, methodology, formal analysis, investigation, writing – original draft. **Oliver Robinson:** conceptualization, methodology, writing – review and editing, supervision, project administration. **Christina M. Lill:** conceptualization, writing – review and editing, supervision. **Anna Matton:** writing – review and editing, supervision. **Raquel Puerta:** data curation, writing – review and editing. **Pilar Sanz:** data curation, writing – review and editing. **Merce Boada:** data curation, writing – review and editing. **Agustín Ruiz:** writing – review and editing. **Lefkos Middleton:** writing – review and editing, supervision, project administration, conceptualization.

## Ethics Statement

Data used in this study were obtained from two independent cohorts: the Alzheimer's Disease Neuroimaging Initiative (ADNI) and the Ace Alzheimer Center Barcelona (Ace). The ADNI study was approved by the Institutional Review Boards of all participating institutions, and written informed consent was obtained from all participants. The Ace cohort study was approved by the Ethics Committee of the Hospital Clinic i Provincial de Barcelona, and all participants provided written informed consent. All procedures were conducted in accordance with the ethical standards of the institutional and/or national research committees and with the 1964 Helsinki Declaration and its later amendments or comparable ethical standards.

## Conflicts of Interest

The authors declare no conflicts of interest related to this work. The research received support from the CSC, but CSC had no influence on the study design, data analysis, interpretation, or publication of results. Additionally, access to data from the ADNI and Ace cohort was granted, though the ADNI and Ace cohort did not provide direct financial support.

## Supporting information


**Table S1:** List of all 190 analytes selected by ADNI and their abbreviations.
**Table S2:** Mean accuracy of ten tests of the classifiers. Each signature included covariates (Age, Sex, *APOE ε4* status). Signature 5 included only covariates.
**Table S3:** Coefficient of the 11 analytes and covariates for AD/CN classification. Participants were classified based on the value closest to their calculated result.
**Table S4:** Coefficient of the 17 analytes and covariates for MCI classification. MCI stable participants were assigned a label of 0, while MCI decliners were assigned a label of 1. Participants were classified based on the value closest to their calculated result.
**Figure S1:** Panel size comparison.

## Data Availability

The data supporting the findings of this study are available from the Alzheimer's Disease Neuroimaging Initiative (ADNI) database (http://adni.loni.usc.edu) upon registration and approval. The Ace cohort data used in this study were provided under a data access agreement and are not publicly available due to privacy or ethical restrictions.
