# Supplementary material for: Plasma Proteomic Signatures for Alzheimer's Disease: Comparable Accuracy to ATN Biomarkers and Cross‐Platform Validation
Source: Ann Clin Transl Neurol. 2025 Oct 13;13(2):354–66. doi: 10.1002/acn3.70227 (PMC12883701; doi:10.1002/acn3.70227)

Supplementary Table S1 List of all 190 analytes selected by ADNI and their abbreviations.

| **Analyte** | **Abbreviation** | **Analyte** | **Abbreviation** | **Analyte** | **Abbreviation** | **Analyte** | **Abbreviation** |
| --- | --- | --- | --- | --- | --- | --- | --- |
| Alpha-1-Microglobulin | A1Micro | Factor VII | Factor VII | Monocyte Chemotactic Protein 2 | MCP-2 | Tissue Inhibitor of Metalloproteinases 1 | TIM1 |
| Alpha-2-Macroglobulin | A2Macro | FASLG Receptor | FAS | Monocyte Chemotactic Protein 3 | MCP-3 | Thrombomodulin | TM |
| Alpha-1-Antichymotrypsin | AACT | Fas Ligand | FasL | Monocyte Chemotactic Protein 4 | MCP-4 | Tenascin-C | TN-C |
| Alpha-1-Antitrypsin | AAT | Fetuin-A | Fetuin-A | Macrophage Colony-Stimulating Factor 1 | MCSF1 | Tumor Necrosis Factor alpha | TNF-alpha |
| Angiotensin-Converting Enzyme | ACE | Fibroblast Growth Factor 4 | FGF-4 | Malondialdehyde-Modified Low-Density Lip | MMLDL | Tumor Necrosis Factor beta | TNF-beta |
| Adrenocorticotropic Hormone | ACTH | Fibroblast Growth Factor basic | FGF-basi | Macrophage-Derived Chemokine | MDC | Tumor Necrosis Factor Receptor-Like 2 | T NFRL2 |
| Adiponectin | Adiponectin | Fibrinogen | Fibrinogen | Macrophage Migration Inhibitory Factor | MMIF | Thrombopoietin | Thrombopoietin |
| Alpha-Fetoprotein | AFP | Ferritin | FRTN | Monokine Induced by Gamma Interferon | MI | TNF-Related Apoptosis-Inducing Ligand Re | TNF-RAILR |
| Agouti-Related Protein | AGRP | Follicle-Stimulating Hormone | FSH | Macrophage Inflammatory Protein-1 alpha | MIP1a | Serotransferrin | Transferrin |
| Angiopoietin-2 | ANG-2 | Granulocyte Colony-Stimulating Factor | GCSF | Macrophage Inflammatory Protein-1 beta | MIP1b | Thyroid-Stimulating Hormone | TSH |
| Angiotensinogen | Angiotensinogen | Growth Hormone | GH | Macrophage Inflammatory Protein-3 alpha | MIP3a | Transthyretin | TTR |
| Apolipoprotein A-I | Apo A-I | Glucagon-like Peptide 1- total | GLP-1 | Matrix Metalloproteinase-1 | MMP-1 | Vascular Cell Adhesion Molecule-1 | VCAM- |
| Apolipoprotein A-II | Apo A-II | Glucagon | Glucagon | Matrix Metalloproteinase-10 | MMP-10 | Vascular Endothelial Growth Factor | VEGF |
| Apolipoprotein A-IV | Apo A-IV | Granulocyte-Macrophage Colony-Stimulatin | GMCS | Matrix Metalloproteinase-2 | MMP-2 | Vitronectin | Vitronectin |
| Apolipoprotein B | Apo B | Growth-Regulated alpha protein | GRO-alph | Matrix Metalloproteinase-3 | MMP-3 | Vitamin K-Dependent Protein S | VKDPS |
| Apolipoprotein C-I | Apo C-I | Glutathione S-Transferase alpha | GST-alp | Matrix Metalloproteinase-7 | MMP-7 | von Willebrand Factor | vWF |
| Apolipoprotein C-III | Apo C-III | Haptoglobin | Haptoglobin | Matrix Metalloproteinase-9 | MMP-9 | Eotaxin-1 | Eotaxin-1 |
| Apolipoprotein D | Apo D | Heparin-Binding EGF-Like Growth Factor | HBEGF | Matrix Metalloproteinase-9- total | MMP-9 | Eotaxin-3 | Eotaxin-3 |
| Apolipoprotein E | Apo E | Chemokine CC-4 | HCC-4 | Myeloid Progenitor Inhibitory Factor 1 | MPIF1 | Erythropoietin | EPO |
| Apolipoprotein H | Apo H | Hepatocyte Growth Factor | HGF | Myeloperoxidase | MPO | Epiregulin | EPR |
| Amphiregulin | AR | Heat Shock Protein 60 | HSP-60 | Myoglobin | Myoglobin | E-Selectin | E-Selectin |
| AXL Receptor Tyrosine Kinase | AXL | T Lymphocyte-Secreted Protein I-309 | I-3 | Neutrophil Gelatinase-Associated Lipocal | NGAL | Endothelin-1 | ET-1 |
| Beta-2-Microglobulin | B2M | Intercellular Adhesion Molecule 1 | ICAM- | Nerve Growth Factor beta | NGF-beta | Fatty Acid-Binding Protein- heart | FABP |
| Brain-Derived Neurotrophic Factor | BDNF | Interferon gamma | IFN-gamma | Neuronal Cell Adhesion Molecule | Nr-CAM | Kidney Injury Molecule-1 | KIM-1 |
| B Lymphocyte Chemoattractant | BLC | Immunoglobulin A | IgA | Osteopontin | Osteopontin | Leptin | Leptin |
| Bone Morphogenetic Protein 6 | BMP-6 | Immunoglobulin E | IgE | Plasminogen Activator Inhibitor 1 | PAI-1 | Luteinizing Hormone | LH |
| Brain Natriuretic Peptide | BNP | Insulin-like Growth Factor-Binding Prote | IGFBP | Prostatic Acid Phosphatase | PAP | Lectin-Like Oxidized LDL Receptor 1 | LOX |
| Betacellulin | BTC | Insulin-like Growth Factor I | IGF-I | Pregnancy-Associated Plasma Protein A | P | Apolipoprotein | Apo A-I |
| Complement C3 | C3 | Immunoglobulin M | IGM | Pulmonary and Activation-Regulated Chemo | PARC | Lymphotactin | Lymphotactin |
| Cancer Antigen 125 | CA-125 | Interleukin-1 alpha | IL-1 alpha | Platelet-Derived Growth Factor BB | PDGF | Monocyte Chemotactic Protein 1 | MCP-1 |
| Cancer Antigen 19-9 | CA-19-9 | Interleukin-1 beta | IL-1 beta | Placenta Growth Factor | PLGF | Testosterone- Total | Testosterone |
| Calbindin | Calbindin | Interleukin-10 | IL-10 | Pancreatic Polypeptide | PPP | Tissue Factor | TF |
| Calcitonin | Calcitonin | Interleukin-11 | IL-11 | Prolactin | PRL | Trefoil Factor 3 | TFF3 |
| CD 40 antigen | CD40 | Interleukin-12 Subunit p40 | IL-12p40 | Progesterone | Progesterone | Transforming Growth Factor alpha | TGF-al |
| CD40 Ligand | CD40-L | Interleukin-12 Subunit p70 | IL-12p70 | Proinsulin- Intact | pM | Transforming Growth Factor beta-3 | TGF-b |
| CD5 | CD5L | Interleukin-13 | IL-13 | Proinsulin- Total | pM | Tamm-Horsfall Urinary Glycoprotein | THP |
| Carcinoembryonic Antigen | CEA | Interleukin-15 | IL-15 | Prostate-Specific Antigen- Free | PSA-f | Thrombospondin-1 | Thrombospondin-1 |
| Chromogranin-A | CgA | Interleukin-16 | IL-16 | Peptide YY | PYY | Epithelial-Derived Neutrophil-Activating | EDNA |
| Creatine Kinase-MB | CK-MB | Interleukin-18 | IL-18 | Receptor for advanced glycosylation end | RAG | EN-RAGE | EN-RAGE |
| Clusterin | CLU | Interleukin-1 receptor antagonist | IL-1r | T-Cell-Specific Protein RANTES | RANTES | Insulin | Insulin |
| Ciliary Neurotrophic Factor | CNTF | Interleukin-2 | IL-2 | Resistin | Resistin | Interferon gamma Induced Protein 10 | IP- 10 |
| Complement Factor H | CFH | Interleukin-25 | IL-25 | S100 calcium-binding protein B | S100-B | Thyroxine-Binding Globulin | TBG |
| Cortisol | Cortisol | Interleukin-3 | IL-3 | Serum Amyloid P-Component | SAP | Thymus-Expressed Chemokine | TECK |
| C-peptide | C-peptide | Interleukin-4 | IL-4 | Stem Cell Factor | SCF | Sortilin | Sortilin |
| C-Reactive Protein | CRP | Interleukin-5 | IL-5 | Secretin | Secretin | Interleukin-8 | IL-8 |
| Connective Tissue Growth Factor | CTGF | Interleukin-6 | IL-6 | Serum Glutamic Oxaloacetic Transaminase | SGOT | Epidermal Growth Factor Receptor | EGFR |
| Cystatin-C | Cystatin-C | Interleukin-6 receptor | IL-6r | Sex Hormone-Binding Globulin | SHBG |  |  |
| Epidermal Growth Factor | EGF | Interleukin-7 | IL-7 | Superoxide Dismutase 1- Soluble | SOD-1 |  |  |

Supplementary Table S2 Mean accuracy of ten tests of the classifiers. Each signature included covariates (Age, Sex, APOE ε4 status). Signature 5 included only covariates.


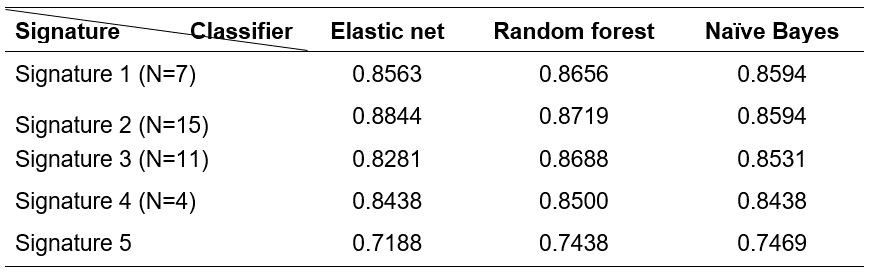


Supplementary Table S3 Coefficient of the 11 analytes and covariates for AD/CN classification. Participants were classified based on the value closest to their calculated result.

| Variable | Coefficient |
| --- | --- |
| Intercept | 0.8804501 |
| sex | -0.000571 |
| age | -0.010135 |
| *APOE ε4* | 0.322872 |
| IL16 | -0.201775 |
| ApoAII | -0.084296 |
| ApoB | 0.1849191 |
| ApoE | -0.090081 |
| Vitronectin | -0.083903 |
| BNP | 0.2498913 |
| IL6r | -0.085611 |
| TTR | -0.112483 |
| PYY | 0.1925992 |
| SGOT | -0.181934 |
| MIP1a | 0.0825047 |

Supplementary Table S4 Coefficient of the 17 analytes and covariates for MCI classification. MCI stable participants were assigned a label of 0, while MCI decliners were assigned a label of 1. Participants were classified based on the value closest to their calculated result.

| Variable | Coefficient |
| --- | --- |
| Intercept | 0.5034435 |
| sex | 0.0070277 |
| age | -0.000142 |
| *APOE ε4* | 0.1234757 |
| ApoCI | 0.0328523 |
| AACT | 0.0503163 |
| ApoAIV | -0.033274 |
| HGF | -0.031666 |
| MIP3a | -0.049505 |
| VEGF | 0.036514 |
| BTC | 0.0087717 |
| CystatinC | 0.0188354 |
| HBEGF | -0.049561 |
| TLSPI3 | -0.056658 |
| MCP4 | -0.003759 |
| ApoCIII | 0.036075 |
| Myoglobin | 0.0839994 |
| PARC | -0.032228 |
| CLU | -0.031745 |
| SCF | -0.016355 |
| Vitronectin | 0.0150791 |

Supplementary Figure S1 Panel size comparison.


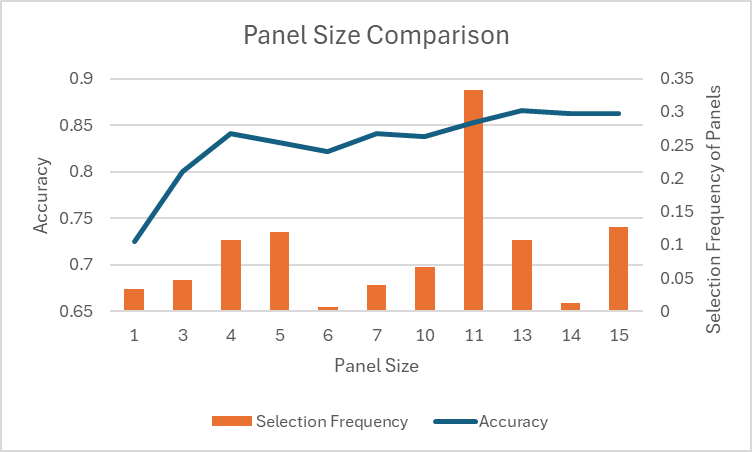

Supplement: Supplementary file 1 — Table S1: List of all 190 analytes selected by ADNI and their abbreviations. Table S2: Mean accuracy of ten tests of the classifiers. Each signature included covariates (Age, Sex, APOE ε4 status). Signature 5 included only covariates. Table S3: Coefficient of the 11 analytes and covariates for AD/CN classification. Participants were classified based on the value closest to their calculated result. Table S4: Coefficient of the 17 analytes and covariates for MCI classification. MCI stable participants were assigned a label of 0, while MCI decliners were assigned a label of 1. Participants were classified based on the value closest to their calculated result. Figure S1: Panel size comparison. [file ACN3-13-354-s001.docx]
